# Identification, heterologous expression and characterization of a dye-decolorizing peroxidase of *Pleurotus sapidus*

**DOI:** 10.1186/s13568-017-0463-5

**Published:** 2017-08-23

**Authors:** Christiane Lauber, Tatiana Schwarz, Quoc Khanh Nguyen, Patrick Lorenz, Guenter Lochnit, Holger Zorn

**Affiliations:** 1 Institute of Food Chemistry and Food Biotechnology, Justus Liebig University Giessen, Heinrich-Buff-Ring 17, 35392 Giessen, Germany; 2 AB Enzymes GmbH, Feldbergstrasse 78, 64293 Darmstadt, Germany; 3 Institute of Biochemistry, Justus Liebig University Giessen, Friedrichstrasse 24, 35392 Giessen, Germany; 4 Bioresources Project Group, Fraunhofer Institute for Molecular Biology and Applied Ecology (IME), Winchesterstrasse 2, 35394 Giessen, Germany

**Keywords:** *Pleurotus sapidus*, Dyp-type peroxidases, White rot, *β*-carotene, Anthraquinone dyes, Lignin degradation

## Abstract

The coding sequence of a peroxidase from the secretome of *Pleurotus sapidus* was cloned from a cDNA library. Bioinformatic analyses revealed an open reading frame of 1551 bp corresponding to a primary translation product of 516 amino acids. The DyP-type peroxidase was heterologously produced in *Trichoderma reesei* with an activity of 55,000 U L^−1^. The enzyme was purified from the culture supernatant, biochemically characterized and the kinetic parameters were determined. The enzyme has an N-terminal signal peptide composed of 62 amino acids. Analysis by Blue Native PAGE and activity staining with ABTS, as well as gel filtration chromatography showed the native dimeric state of the enzyme (115 kDa). Analysis of the substrate range revealed that the recombinant enzyme catalyzes, in addition to the conversion of some classic peroxidase substrates such as 2,2′-azino-bis(3-ethylthiazoline-6-sulfonate) and substituted phenols like 2,6–dimethoxyphenol, also the decolorization of the anthraquinonic dye Reactive Blue 5. The enzyme also catalyzes bleaching of natural colorants such as *β*-carotene and annatto. Surprisingly, *β*-carotene was transformed in the presence and absence of H_2_O_2_ by rPsaDyP, however enzyme activity was increased by the addition of H_2_O_2_. This indicates that the rPsaDyP has an oxidase function in addition to a peroxidase activity. As a consequence of the high affinity to the characteristic substrate Reactive Blue 5 the rPsaDyP belongs functionally to the dyp-type peroxidase family.

## Introduction

Heme peroxidases have been classified into various superfamilies according to their functional and structural properties (Morgenstern et al. 2008). According to the classification of Welinder (1992) DyP-type peroxidases were assigned to Class II of the plant-peroxidase superfamily. This class includes the secretory fungal peroxidases and is characterized by a wide homogeneity; for example the manganese peroxidases (MnP), lignin peroxidases (LiP) and the versatile peroxidases (VP) all belong to this class (Lundell et al. 2010; Martíınez 2002). All class II peroxidases are extracellular and contain heme as the prosthetic group (Poulos 2010; Welinder 1992). The DyP-type peroxidases, however, show no homology to any other known peroxidase families. They possess a unique characteristic that differentiates them from other heme peroxidases and thus they consequently form their own superfamily (EC 1.11.1.19) among the heme-peroxidases. Recently Zámocký et al. (2009, 2015) have suggested a new classification based on the overall fold, the structure of the active center and enzymatic activity. DyP-type peroxidases are now consequently allocated to the peroxidase-cyclooxygenase superfamily that is characterized by ferredoxin-like folding of the *β*-sheet structure and represents part of the very large α/β-barrel structure superfamily. The first indication of the existence of this peroxidase type was discovered by Kim et al. (1995). The first enzyme of this family (Bad DyP) was extracted from the fungus *Bjerkandera adusta* and was consequently purified and characterized (Kim and Shoda 1999a). In the meantime DyP-type peroxidases have not only been discovered in Basidiomycota, but also in Ascomycetes and bacteria (Hofrichter et al. 2010). This also implies that these peroxidases have a common origin before the division of the domains (Sugano 2009). The classical DyP from *Bjerkandera adusta* is the most completely characterized member of the family of DyP-type peroxidases. The term dye decolorizing peroxidase “DyP” presently describes a more polyphyletic group of enzymes (Ahmad et al. 2011). A subdivision of the peroxidases into three groups (P, I, V) has recently been suggested for the classification of DyP-type peroxidases (Yoshida and Sugano 2015).

Ten DyP-type peroxidases from fungi have been thus far characterized, and only those from *B. adusta* und *A. auricula*-*judae* have been characterized from a structural and mechanical perspective (Linde and Coscolñas 2014; Linde et al. 2015a; Strittmatter et al. 2013; Sugano 2009; Yoshida et al. 2011, 2012). DyP–type peroxidases possess a unique H_2_O_2_-binding site that differs from those of other peroxidases (Sugano et al. 1999). Whereas in the enzymes of the superfamily of plant peroxidases the rearrangement of the protons of H_2_O_2_ is typically mediated by the distal histidine this is accomplished by an aspartate in DyP-type peroxidases (Sugano et al. 2007).

DyP-type peroxidases are catalytically very versatile and stable (Pühse et al. 2009). They possess a high redox potential (1.1–1.2 V) so that numerous substrates can serve as electron donors (Liers et al. 2013a). DyP-type peroxidases are therefore able to oxidize a wide spectrum of complex dyes, in particular xenobiotic anthraquinone derivatives as well as typical peroxidase substrates such as ABTS (2,2′-azino-bis(3-ethylthiazoline-6-sulfonate)) and phenols (Sugano 2009). Certain Dyp-type peroxidases cleave *β*-carotene and other carotenoids, non-phenolic bonds such as veratryl alcohol and the β-*O*-4 lignin model dimer adlerol (Liers et al. 2010).

*Pleurotus sapidus* is a Basidiomycete of the family Pleurotaceae (oyster mushrooms) belonging to the white-rot fungi. It grows on wood (pillar fungus). White-rot fungi are the most efficient lignin destruents and are able to fully decompose complex polymers (Rajarathnam et al. 1998). In addition, *P. sapidus* is able to decompose natural pigments such as *β*-carotene (Schüttmann et al. 2014). Numerous enzymes that are involved in lignocellulose decomposition have been identified in the secretome of *P. sapidus.* For example, the fungus secretes cellulases, hemicellulases, peptidases, esterases, laccases and in particular peroxidases into the culture medium (Zorn et al. 2005).

As a result of their wide substrate spectrum DyP-type peroxidases are interesting tools for biotechnological processes used in the decomposition of xenobiotic anthraquinone derivatives in sewage and soil (Husain 2006; Sugano et al. 2007) and are already finding use in the food industry. For example, the DyP-type peroxidase MsP1 from *M. scorodonius* (Scheibner et al. 2008), marketed as MaxiBright^®^ (DSM, Heerlen, the Netherlands), is used to whiten whey for cheese production. The enzyme thus represents an alternative to chemical bleaches (Szweda et al. 2013).

In the present report we publish a cDNA sequence that codes for a DyP-type peroxidase from *Pleurotus sapidus*. Only two reports have been published on the DyPs from Pleurotus fungi (Faraco et al. 2007; Fernández-Fueyo et al. 2015). The enzyme was efficiently expressed heterologously in *Trichoderma reesei*, purified and biochemically characterized. Homology studies allow the identification of important catalytic amino acids and potential substrate oxidation sites.

## Materials and methods

### Chemicals

Chemicals and reagents used were obtained from Carl Roth (Karlsruhe, Germany), Sigma (Neu-Ulm, Germany) or Merck (Darmstadt, Germany). Chemicals and materials for electrophoresis were from Serva and Bio–Rad.

### Strains and culture methods

*Pleurotus sapidus* (DSM 8266) was obtained from the German Collection of Microorganisms and Cell Cultures (DSMZ, Braunschweig, Germany). The Basidiomycete was cultivated in standard culture medium (30 g L^−1^ d–(+)–glucose·H_2_O, 4.5 g L^−1^ l-asparagine·H_2_O, 1.5 g L^−1^ KH_2_PO_4_, 0.5 g L^−1^ MgSO_4_·H_2_O, 3.0 g L^−1^ yeast extract, 1 mL L^−1^ trace element solution: 5 mg L^−1^ CuSO_4_·5H_2_O, 80 mg L^−1^ FeCl_3_·6H_2_O, 90 mg L^−1^ ZnSO_4_·7H_2_O, 30 mg L^−1^ MnSO_4_·H_2_O, and 0.4 g L^−1^ EDTA; pH 6.0) for 10 days (24 °C, 150 rpm, 25 mm shaking diameter).

*Escherichia coli* (TOP10) was obtained from Invitrogen (Karlsruhe, Germany) and was used for vector propagation. TSS competent cells were transformed by heat shock treatment (2 min, 42 °C) according to the standard protocols. Recombinant cells were cultivated in sterile LB medium (10 g L^−1^ tryptone, 5 g L^−1^ yeast extract, 10 g L^−1^ NaCl) with 150 mg L^−1^ ampicillin used as selection marker (37 °C, 225 rpm).

### cDNA-synthesis

To isolate the RNA the mycelium of a *P. sapidus* submersion culture was harvested on culture day 5 and 100 mg was disintegrated by grinding in liquid nitrogen. Isolation of the total RNA was performed with RNeasy™ Plant Mini Kits (Qiagen, Hilden, Germany) according to the manufacturer’s instructions. The quality of the RNA was verified by agarose electrophoresis and ethidium bromide staining. A cDNA library of *P. sapidus* was produced using the isolated RNA as template. cDNA synthesis was performed with help of the SMART™ PCR cDNA Synthesis Kits (Clontech Laboratories Inc., Saint-Germain-en-Laye, France). SuperScript^®^ II Reverse Transcriptase (Invitrogen, Darmstadt, Germany) was used for first-strand synthesis. Amplification of a coding DyP-type peroxidase sequence of *P. sapidus* from the cDNA library was performed by PCR. The primers DTP_for58 (5′-ATGCGCTGGTGGACTACC-3′) and DTP_rev54 (5′-TTAAGCAGCGATTTTGTGC-3′) were derived from a homologous Dyp-type peroxidase sequence from the genome (DOE-JGI) of *Pleurotus ostreatus*. Amplification of the specific cDNA occurred in an Alpha SC PCR Thermocycler (Analytik Jena, Jena, Germany). The following PCR protocol was followed: 50 ng template, 5× PCR-Puffer including dNTP’s (Qiagen), forward and reverse primer 50 pmol, 1.25 U HotStar HiFidelity DNA-Polymerase (Qiagen) ddH_2_O ad 50 µL, 95 °C 5 min–95 °C 1 min, 51 °C 1 min, 72 °C 2 min, 40 cycles–72 °C 5 min. PCR products were separated electrophoretically (1.2% (w/v) agarose gel), subsequently isolated from the gel using NucleoSpin Extract II–Kits (Machery-Nagel, Düren, Germany) and finally ligated (Topo TA-Cloning^®^ Kit, Invitrogen) in the vector pCR2.1–TOPO^®^ (Invitrogen). The plasmid DNA was replicated in *E.* *coli* TOP10 cells (Invitrogen), isolated from the cells and purified (NucleoSpin^®^ Plasmid DNA Purification, Machery-Nagel). Sequencing of the cloned cDNA was performed by MWG Eurofins (Ebersberg, Germany).

### Enzyme production

For recombinant production of the peroxidase the codon usage of the gene was adapted to the host organism *Trichoderma reesei* (a derivative of RUT C30; Peterson and Nevalainen 2012) and an expression cassette was constructed from the adapted sequence. The expression of peroxidase took place under the control of the cbhl promotor and terminator. Efficient production of the peroxidase was achieved by optimization of the fermentation process under fed-batch conditions (pH5.5, 28 °C, 160 h) in Monosaccharide medium (7% monosaccharide, 4% agricultural waste stream derived N-source, 0.7% (NH_4_)_2_SO_4_, 0.3% KH_2_PO_4_).

### Determination of enzyme activity

Enzyme activity was determined photometrically using a temperature controlled multi-mode plate reader (Synergy TM 2, BioTek Instruments GmbH, Bad Friedrichshall, Germany) or alternatively in a UV/Vis spectrophotometer (SPECORD^®^ 50, Analytik Jena AG, Jena, Germany). Reactions were initiated by addition of the enzyme. Enzyme activity was measured over a period of 10 min at 25 °C at the appropriate wavelength for the substrate. One unit (1 U) is defined as the amount of enzyme that converts 1 µmol substrate per minute. Various H_2_O_2_ concentrations (0–1250 µM, enzyme concentrations (0.27–54 nM) and substrate concentrations were used to determine the enzyme activity.

The activity of rPsaDyP vs ABTS was determined in 100 mM sodium acetate buffer at pH 3.8 and a final H_2_O_2_ concentration of 125 µM. Production of the ABTS cation radical was studied according to Eggert et al. (1996) at 420 nm (ε_420_ 36,000 L mol^−1^ cm^−1^).

Oxidation of DMP and guaiacol was followed in 50 mM sodium acetate buffer at pH 4.5 at a final H_2_O_2_ concentration of 62.5 µM as based on the formation of the dimeric quinone derivate at 469 nm (ε_469_ 27,500 L mol^−1^ cm^−1^ referred to the substrate) according to Saparrat et al. (2002) or tetraguaiacol at 470 nm (ε_470_ 26,600 L mol^−1^ cm^−1^) according to Koduri and Tien (1995).

Oxidation of Reactive Blue 5 dye (Rblue 5) was performed in 100 mM sodium acetate buffer at pH 4.0 and a final H_2_O_2_ concentration of 31.2 µM. Degradation of the substrate was determined at 600 nm (ε_600_ 8000 L mol^−1^ cm^−1^) according to Sugano et al. (2006).

Oxidation of the azo dye Reactive Black 5 (RBlack 5) was determined in 50 mM sodium acetate buffer at pH 4.0 with 62.5 µM H_2_O_2_ at 598 nm (ε_598_ 37,200 L mol^−1^ cm^−1^) according to Sugano et al. (2006).

A *β*-carotene stock solution was prepared as described by Pühse et al. (2009). Measurement of the substrate degradation was performed at 450 nm (ε_450_ 95,000 L mol^−1^ cm^−1^) according to Ben Aziz et al. (1971) in 50 mM sodium acetate buffer at pH 3.5 with a final H_2_O_2_ concentration of 125 µM. To assess the purified enzyme’s oxygenase activity in the absence of H_2_O_2_, 100 mL sodium acetate buffer (100 mM, pH 3.8) was purged with oxygen through a glass frit (pore size 3, pore diameter 16–40 μm) for 30 min. For control experiments in the absence of H_2_O_2_ and oxygen, 100 mL of the buffer was sonicated for 60 min, and residual oxygen was removed by purging with nitrogen. The assays were performed with enzyme concentrations of 270, 360, and 540 nM, and the concentration of the substrate was 26 µM.

As further examples of natural dyes annatto (aqueous alkaline extract), the principle component of which is norbixin, and bixin were used as substrates. Preparation of the bixin stock solution was analogous to that of *β*-carotene (using 15 mg bixin). Conversions took place in 50 mM sodium acetate buffer at pH 6.0 or 3.5 with a final H_2_O_2_ concentration of 125 µM. Degradation of the dye was observed at 452 nm (ε_452_ 108,400 L mol^−1^ cm^−1^, Scotter et al. 1998) or 465 nm (ε_465_ 136,100 L mol^−1^ cm^−1^, Hülsdau 2007).

Samples of heat inactivated (95 °C, 10 min) enzyme served as negative controls.

### Purification of the DyP-type peroxidase by fast protein liquid chromatography (FPLC)

Purification was carried out in a cold room at 4 °C. Chromatographic separation of the proteins was performed using a BioLogic DuoFlow™ (Biorad) FPLC–System with fraction collector (BioLogic BioFrac™ fraction collector, Biorad). Proteins were detected at 280 nm. The DyP-type peroxidase was purified in a two-stage process by hydrophobic interaction chromatography and ion exchange chromatography. The culture supernatant was concentrated (Macrosep^®^, 10 kDa cutoff, Pall, Dreieich, Germany) and transferred to 50 mM sodium acetate buffer at pH 4.0 with 1 M (NH_4_)_2_SO_4_. In the first stage the concentrated culture supernatant was purified on a column with phenyl-Sepharose-matrix (HiPrep Phenyl FF (high sub) 16/10, GE Healthcare Bio–Sciences AB, Uppsala, Sweden). The Starting buffer used was 50 mM sodium acetate buffer pH 4.0 with 1 M (NH_4_)_2_SO_4_ and proteins were eluted over a gradient of 0 to 58% (40 mL) and from 58 to 100% (30 mL) sodium acetate buffer pH 4.0. Flow rate was 3 mL min^−1^ and fractions with a volume of 2 mL were collected. Protein containing fractions were tested and active fractions pooled for the second purification step. They were then concentrated, transferred to 50 mM sodium acetate buffer pH 4.0 and loaded onto a column with SP Sepharose Fast Flow Matrix (XK16/20, 25 mL column volume, GE Healthcare Bio–Sciences AB). Elution was performed on a gradient of 0–10% (30 mL) and from 10 to 100% (30 mL) 50 mM sodium acetate buffer pH 4.0 with 1 M NaCl at a flow rate of 3 mL min^−1^. Active fractions of the second purification stage were pooled, concentrated, aliquoted, shock frozen in liquid nitrogen and stored at −80 °C.

### Enzyme characterization

Protein concentration was determined according to Bradford (1976) using the dye solution Roti^®^ Nanoquant (Roth, Karlsruhe, Germany) with bovine serum albumin or lyophilized DyP as standard.

UV/Vis spectra of the purified rPsaDyP were recorded at 250 nm to 800 nm in a NanoPhotometer (Pearl, Implen GmbH, Munich, Deutschland).

Molecular mass determination of the DyP-type peroxidase was performed using SDS–PAGE (Mini–Protean^®^ TetraSystem, BioRad) according to Laemmli. Separation of the proteins took place in a 6% stacking and a 12% separation gel. Protein bands were visualized using colloidal Coomassie staining (Kang et al. 2002). Size determination was performed with the help of PageRuler™ Unstained Proteinladder (Fermentas, St. Leon–Rot, Germany). For N-deglycosylation samples were incubated with PNGase F (NEB, Ipswich, USA) and treated following the manufacturer’s instructions.

The size of the native proteins was determined by gel filtration chromatography (GFC) and Blue Native PAGE. For GFC the protein was loaded onto a Superdex 200 10/300 GL column (GE Healthcare) and eluted with a 50 mM sodium acetate buffer pH 4.0 with 250 mM NaCl and 0.005% Triton X-100 at a flow rate of 0.5 mL min^−1^. For Blue Native PAGE precast gels from Serva (SERVAGel™ N Native Starter Kit, Serva, Heidelberg, Germany) and the PerfectBlue^®^ Double Gel System Twin S of Peqlab were used. For isoelectric focusing (IEF) the same electrophoresis system was used (precast vertical gels and IEF marker 3–10, Liquid Mix, Serva). Visualization of the protein bands was performed as described above. In addition, specific staining of heme- and metal enzymes was performed with 3,3′,5,5′-tetramethylbenzidine (TMB) (1.5 mg mL^−1^ in methanol) modified according to Thomas et al. (1976) and Henne et al. (2001). Native electrophoresis was followed by activity staining with ABTS as substrate. For this the gel was equilibrated for 5 min in 100 mL freshly prepared stain solution (5 mM ABTS in 50 mM sodium acetate pH 4.0) on an orbital shaker. Subsequently, 150 μL 3% H_2_O_2_ (final concentration 130 μM) was added and shaking continued until a distinct staining was obtained. Gels were then rinsed with pure water and documented.

### Influence of pH value

The optimum pH of the purified rPsaDyP was determined in McIlvaine–Puffer (pH 2.2–7.5, McIlvaine, 1921), 50 mM sodium acetate buffer (pH 3.0–6.0) and 100 mM sodium tartrate buffer (pH 2.0–5.5) with ABTS as substrate. The pH stability was determined by diluting the DyP-type peroxidase in sodium acetate buffer at a pH range of 3.0–6.0 (0.5 steps). To avoid altering the pH value too drastically for the measurement the samples were again diluted 1:10 with 100 mM sodium acetate buffer pH 3.8 (measurement buffer). Rest activity was measured after 0.5, 1, 2 and 24 h storage at 4 °C using ABTS as substrate and values were compared to the initial activity.

### Effect of temperature

To determine the optimum pH for the purified rPsaDyP the enzyme was initially incubated for 4 min in 50 mM sodium acetate buffer at pH 3.8 at various temperatures (15–75 °C). The rate of turnover was then measured at the corresponding temperature in a photometer with temperature regulated cuvette.

### Determination of kinetic parameters

The apparent value of the Michaelis–Menten constant (*K*_*m*_) and catalytic constant (*k*_cat_) of the purified rPsaDyP were determined for ABTS, DMP, guaiacol and RBlue 5. Substrate turnover took place at 30 °C in 6 replicate preparations. The initial rate was determined with constant enzyme concentration, varying substrate concentrations and constant cosubstrate concentration. Measurements were taken in parallel with varying H_2_O_2_ concentrations in order to determine the H_2_O_2_ concentration that no longer had a limiting effect on the activity. The following substrate concentrations were chosen for determination of the kinetic parameters: ABTS 15–1500 µM, DMP 250–15,000 µM, guaiacol 500–15,000 µM, RBlue 5 11–300 µM. The initial rate (*ν*_*0*_) was plotted linear to the substrate concentration in a graph (Cornish-Bowden diagram). Using the software Origin (OriginPro 8.6G) a saturation hyperbola was adapted through a non-linear regression in order to obtain values for *K*_*m*_ and *ν*_max_. The catalytic constant *k*_cat_ was determined using the following equation, whereby E represents the enzyme concentration applied:

$$k_{{cat}} = \frac{{v_{{\max }} }}{{[E]}}$$

## Results

### Identification of the coding sequence of DyP-type peroxidase

**Fig. 1:**
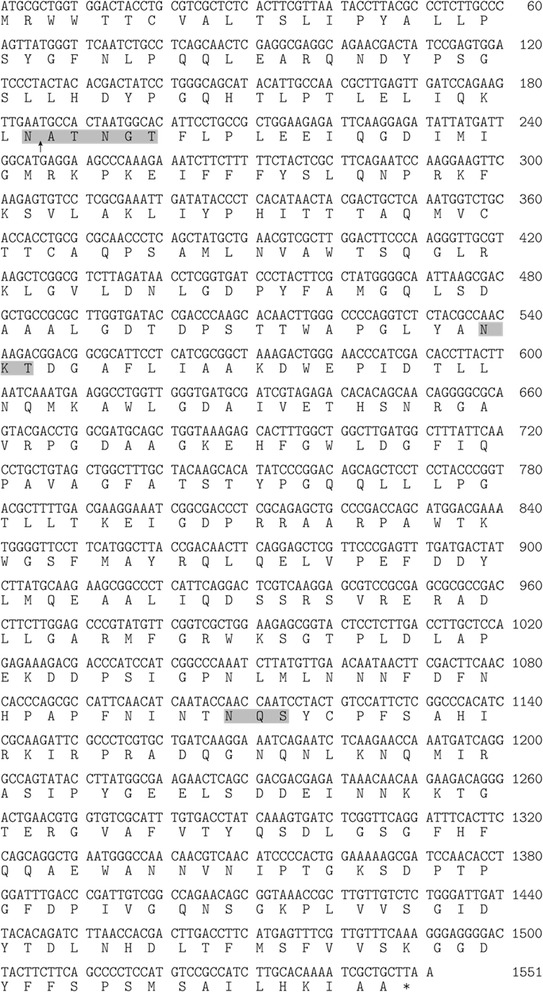
Nucleotide- and derived amino acid sequence of a DyP-type peroxidase from *Pleurotus sapidus* (LN830264). Predicted N-glycosylation sites are highlighted; a hypothetical cleavage site after 62 amino acids is marked with an *arrow*

The 1551 bp full-length cDNA of a DyP-type peroxidase gene of *Pleurotus sapidus* (strain 8266) was amplified. This gene codes for a protein of 516 AA (Fig. 1, Accession Number LN830264), a theoretical molecular weight of 57.1 kDa and a calculated isoelectric point (pl) of 5.73 (ExPASy ProtParam). The ORF exhibits 4 potential *N*-glycosylation sites and a potential *O*-glycosylation site (NetNGly 1.0, NetOGly3.1).

With the identification of putative conserved domains the enzyme is assigned to the *dye decolorizing peroxidase* superfamily (EMBL-EBI, InterProScan). In addition, the structural motif GxxDG that is present in all members of the DyP-type peroxidase family was found in the sequence. The translated amino acid sequence shows a high degree of identity (95%) to a DyP-type peroxidase from *Pleurotus ostreatus* (ID EMBL CAK55151.1). Furthermore, the amino acid sequence exhibits an identity of 42–43% with DyP-type peroxidases from *B. adusta* (Accession Number: BAA77283.1), *M. scorodonius* (Accession Number: B0BK71.1) and *A. auricula*-*judae* (Accession Number: AFJ79723).

### Structure and sequence based analysis

A structural homology model of the DyP-type peroxidase from *P. sapidus* was calculated based on the x-ray crystal structure of DyP-type peroxidase from *A. auricula*-*judae* (PDB-ID 4AU9B). The structures exhibit a homology of 44%.

**Fig. 2:**
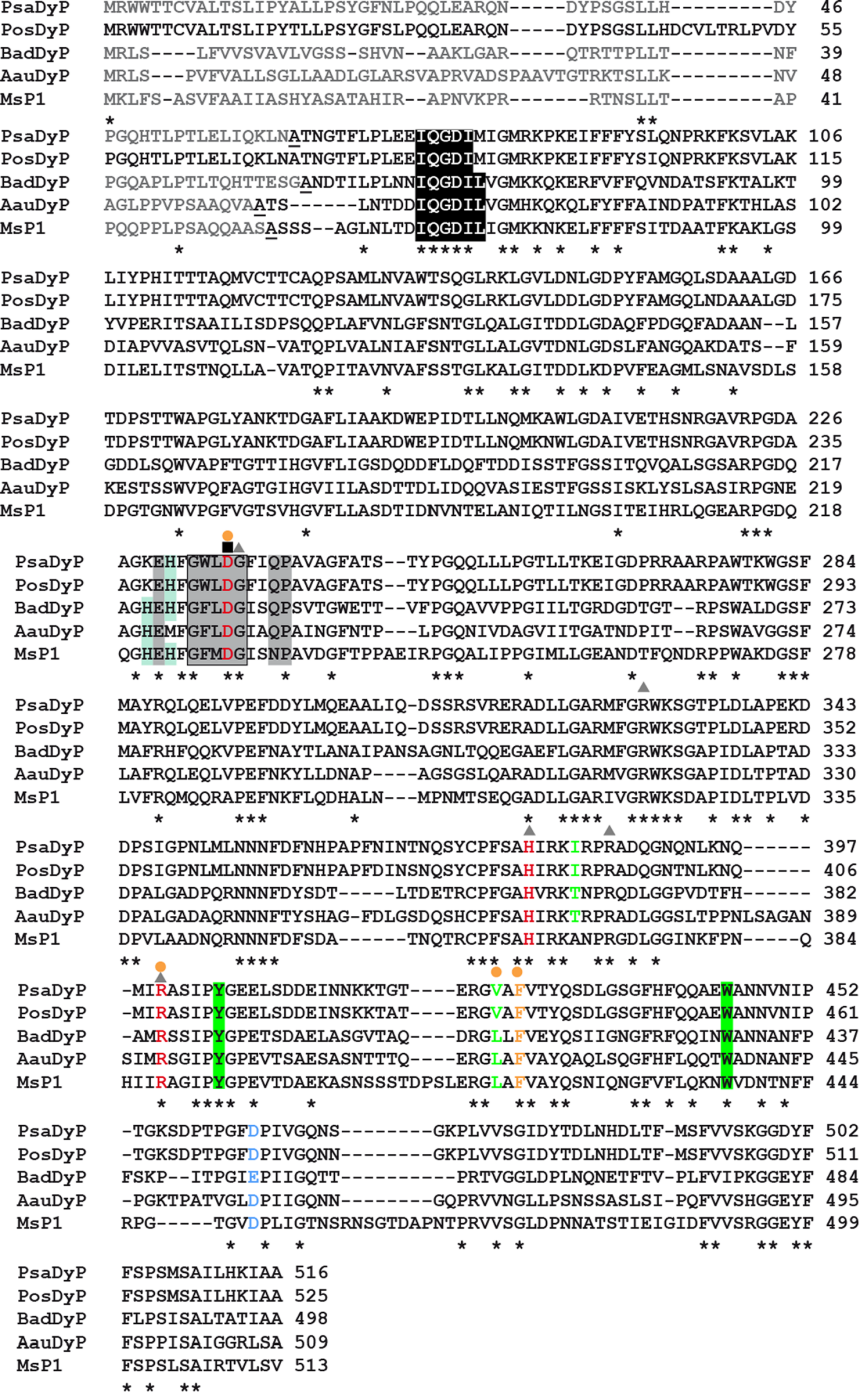
Alignment of various DyP-type peroxidase sequences from *P. sapidus* (PsaDyP), *P. ostreatus* (PosDyP, NCBI: CAK55151), *B. adusta* (BadDyP, NCBI: BAA77283), *A. auricula*-*judae* (AauDyP, NCBI: AFJ79723) and *M. scorodonius* (MsP1, NCBI: BOBK71). Conserved amino acids (*); the characteristic GxxDG motif is enclosed in a frame; remnants of the H_2_O_2_ binding site (*circle*); E391 (*blue*); conserved residues of the heme binding site (*gray background*); potential heme binding residues (*triangle*); exposed amino acids of a potential LRET (long range electron transfer, *green* background); remnants involved in a potential LRET (*green*); N-terminal conserved amino acid sequences (*white*); conserved histidine residues (H164 and H166, *blue background*); signal sequences (*gray*); N-terminal amino acids are *underlined*

The calculated structural model possesses a helical basic structure with two domains (proximal N-terminal, distal C-terminal domains), that enclose the central cofactor heme. A prominent motif made up of anti-parallel *β*-sheet structures that, together with the *α*-helices, exhibit a ferredoxin-like folding is present on the distal side of the heme. With the help of this structure homology model it was possible to identify important functional amino acid residues (Fig. 2). The access channel to the heme of the catalytic channel forms a predominantly hydrophobic channel through which the substrate reaches the H_2_O_2_ binding pocket (Yoshida et al. 2011). Three of the remaining residues of the H_2_O_2_ binding pocket are also conserved in rPsaDyP, whereby in the DyP-type peroxidases from *P. sapidus* and *P. ostreatus* leucine is exchanged for valine (rPsaDyPV363). The catalytic residues of D174—part of the conserved GxxDG motif—and R338 on the proximal side H317 as fifth ligand of heme were identified. These residues are involved in the activation of the enzyme (formation of compound I) by heterolytic cleavage of H_2_O_2_. Based on the alignment (Fig. 2) it was shown that E391, which forms a hydrogen bond with the fifth ligand of heme (H317), thus stabilizing compound I (Sugano et al. 2007), is exchanged for an aspartate (rPsaDyP D401) in other Dyp-type peroxidases. The arginine 267 and 324 hydrogen bonds with the propionate residues of the heme form additional ligands. The conserved amino acid residues Y343 and W383 that were exposed to the solvent are found on the surface. These serve as oxidation sites for large substrates and may be elements of a LRET (long range electron transfer) (Liers et al. 2013a; Strittmatter et al. 2013).

### Heterologous expression and purification

**Fig. 3:**
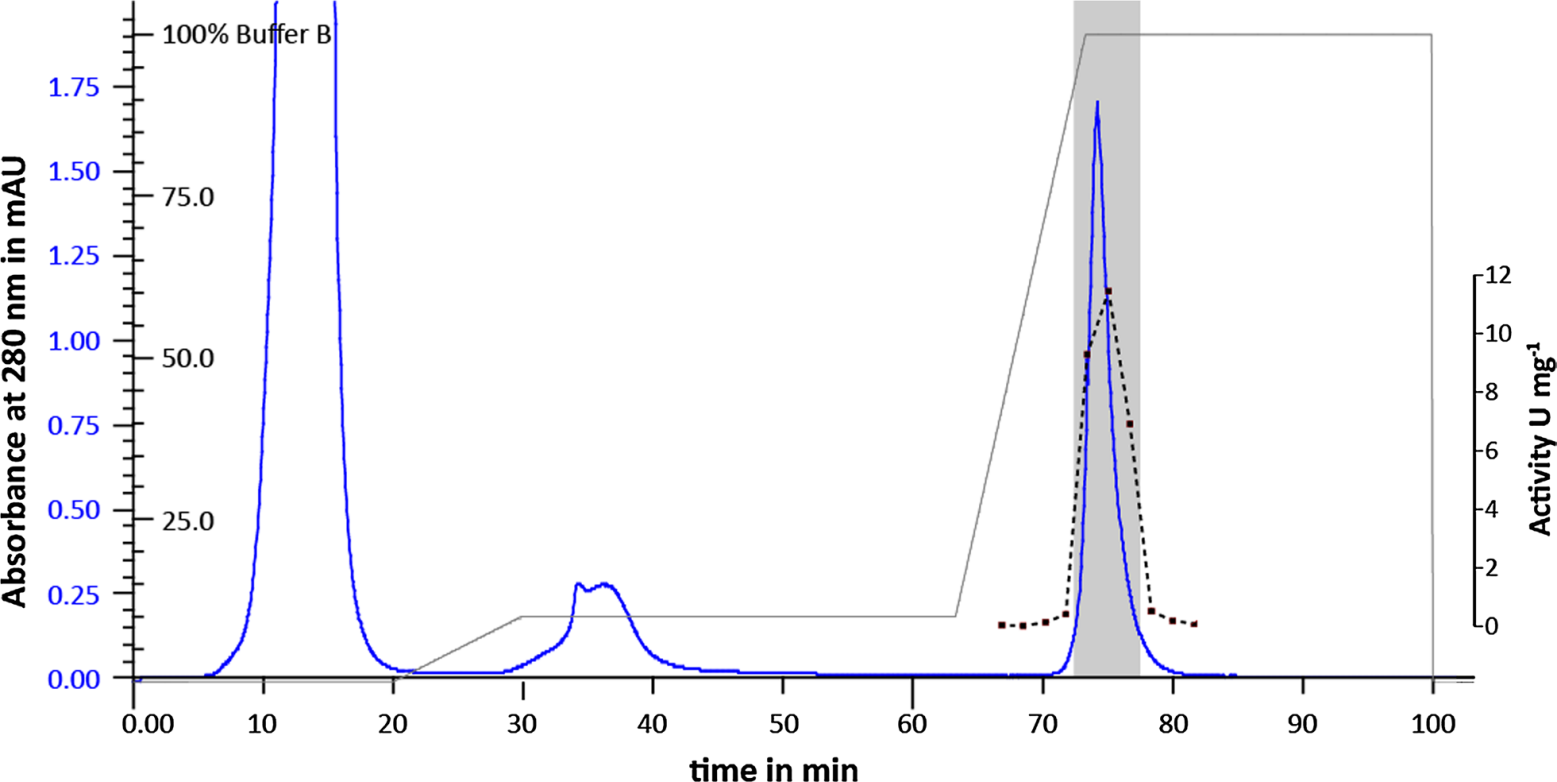
Cation-exchange chromatography as second purification step using an SP Sepharose FF column; ABTS oxidizing enzyme activity was detected in the area with a *gray* background; UV absorption in AU at 28 nm (*blue line*), detected activity (*dashed line*), buffer concentration (50 mM sodium acetate buffer pH 4.0 with 1 M NaCl) in  % (*light gray line*)

For purification and characterization peroxidase positive transformants were cultivated in large scale (XL) under conditions that yield active protein in the culture supernatant. After 160 h cultivation an activity of 55,000 U L^−1^ in relation to the substrate ABTS was achieved and the supernatant containing the peroxidase was harvested. After concentration of the culture supernatant (crude enzyme) by a factor of 5 the Dyp-type peroxidase was purified in a two-stage sequential procedure using FPLC with hydrophobic interaction chromatography and ion exchange chromatography. The DyP-type peroxidase was purified to apparent homogeneity. After the second purification step the active fractions eluted in a single, discreet peak (Fig. 3). Following the two-stage purification an electrophoretic homogeneity of DyP was achieved with a yield of 2.9 ± 0.1 g L^−1^ and an increase in Reinheitszahl from 0.2 to 1.1.

### Characterization of rPsaDyP

**Fig. 4:**
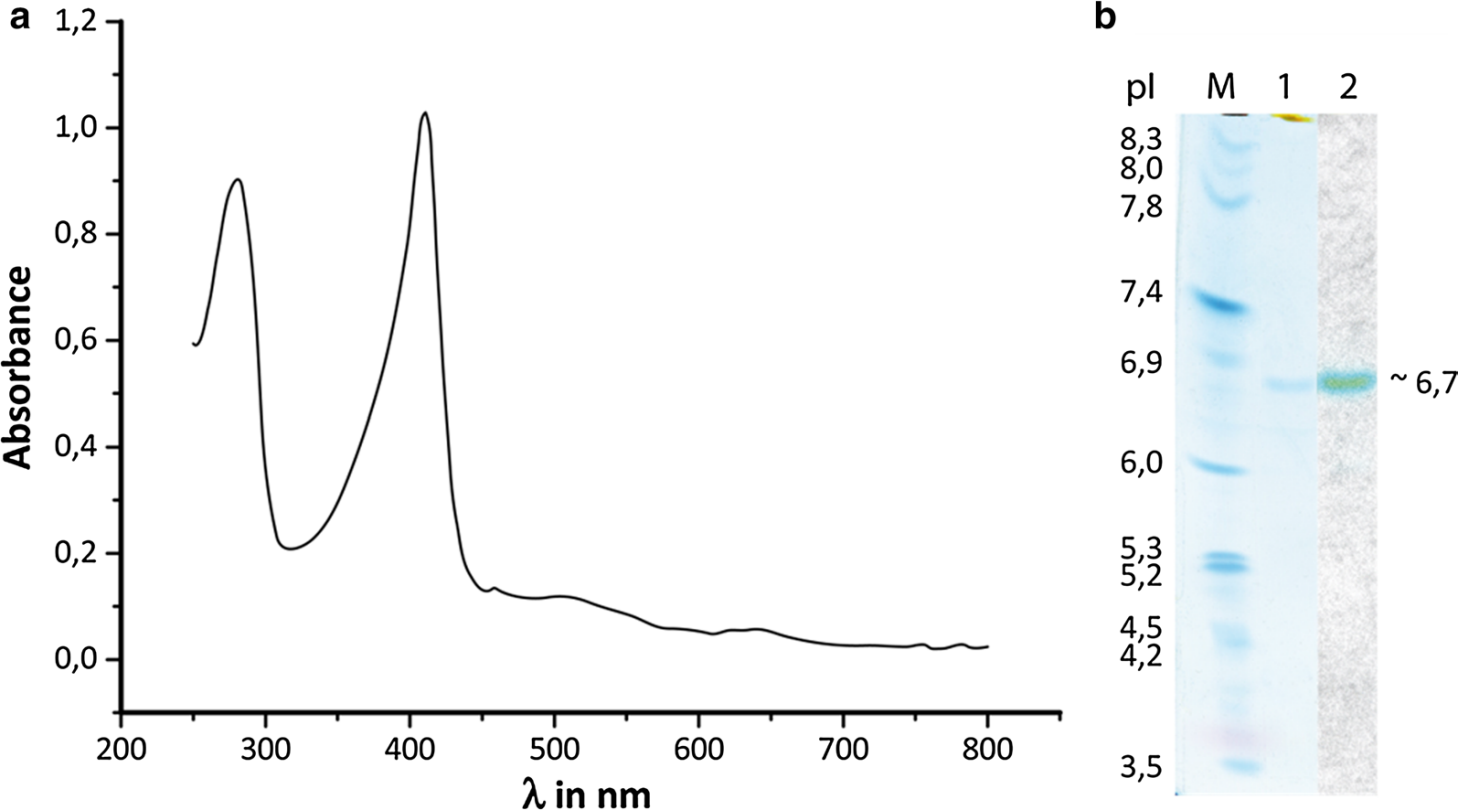
**a** UV–Vis absorption spectrum of the purified rPsaDyP showing the Soret band at 409 nm and two additional maxima in the region of 510 and 640 nm; **b** isoelectric focusing of purified rPsaDyP, stained with colloidal Coomassie (1) and specific staining for heme/metal enzymes with TMB (2); M IEF Marker 3–10

The purified enzyme showed a typical heme-enzyme red coloring and a maximum absorbance λ_max_ = 409 nm and two further maxima at 510 and 640 nm (Fig. 4). A molecular weight of 57.6 kDa was determined by SDS-PAGE. Isoelectric focusing (IEF) showed a pl of 6.7 (Fig. 4).

### Determination of the native conformation

The native conformation of the DyP was established by *Blue Native* PAGE and gel filtration chromatography. By electrophoresis a molecular weight of 115 kDa was ascertained for the native, active enzyme and the retention time of the peaks in gel filtration chromatography indicated an apparent molecular weight of 122 kDa. The molecular weights indicated by these two methods were comparable and approximately double the value of the apparent molecular weight of rPsaDyP under denaturing conditions (57.6 kDa). This suggests that the quaternary structure of the native enzyme is a dimer.

Four potential N-glycosylation sites were predicted in the amino acid sequence. The protein was therefore treated with PNGase F in order to remove any possible oligosaccharides from the N-glycosylation sites. After treatment with the glycosidase rPsaDyP showed a lower apparent molecular weight in SDS-PAGE than the untreated samples (52.8–57.6 kDa). This indicates that rPsaDyP is glycosylated by the host organism. From the difference in molecular weights a degree of glycosylation of ~9% was calculated.

### N-terminus

The detected apparent molecular weight of the deglycosylated rPsaDyP is lower than the theoretical weight that was calculated from the primary sequence. This suggests that the protein is processed in the host organism. Therefore, the N-terminus of the recombinant enzyme was determined by Edman degradation. The first 12 residues of the N-terminal amino acid sequence of the purified enzyme (AT(N)GTFLPLEEI) were identified. According to this the enzyme has a signal peptide 62 amino acids long and the mature protein a total length of 454 amino acids.

### Catalytic properties of rPsaDyP

#### pH- and temperature optimum

**Fig. 5:**
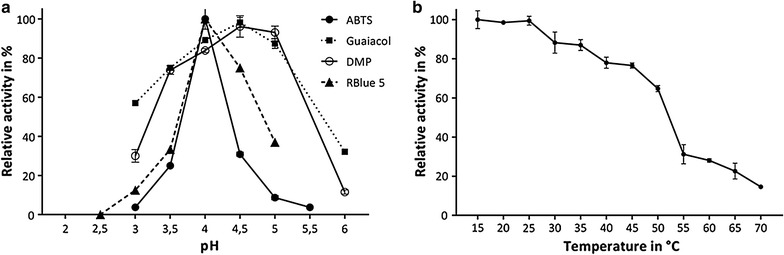
**a** Optimum pH value for oxidation of ABTS, DMP and guaiacol by rPsaDyP in 50 mM sodium acetate buffer or Rblue 5 in 100 mM sodium tartrate buffer. **b** Effect of temperature on the activity of rPsaDyP with ABTS as substrate

Before the kinetic constants were determined the optimal pH for oxidation of the substrate investigated was identified (Fig. 5). The pH-optimum for the oxidation of ABTS and Rblue 5 was found to be between 3.5 and 4.0, for the reaction with phenolic substrates the pH-optimum was higher (pH 4.5). In addition, the enzyme activity for the substrates was determined under varying H_2_O_2_ concentrations (0–1250 µM). With ABTS as substrate the peroxidase activity fell significantly when the H_2_O_2_ concentration rose above 0.125 mM, indicating that the enzyme is inhibited by H_2_O_2_. Maximum reaction rates depending upon substrate tested reached values between 31.2 and 125 µM (31.2 µM: RBlue 5, 62.5 µM: DMP, guaiacol, RBlack 5; ABTS, Annatto, Bixin, *β*-carotene: 125 µM).

The enzyme showed maximum activity over a temperature range of 15–30 °C. To determine the thermal stability residual activity was detected after a 5 min incubation of the enzyme. At temperatures over 70 °C the enzyme completely lost activity. A 5–min T_50_–Wert of 53 °C was determined from the residual activity of the enzyme. Under assay conditions the enzyme showed a 75% residual activity after 2 h and after 24 h 40% of the initial activity was still present.

#### Catalytic properties

The apparent kinetic constant for rPsaDyP (expressed in *T. reesei* as active protein) was determined for ABTS, RBlue 5, DMP and guaiacol as shown in Table 1. The peroxidase efficiently oxidized the low redox potential dye ABTS. In addition, rPsaDyP catalyzed the degradation of the dye Rblue5, a characteristic DyP substrate. The enzyme binds this substrate at high affinity (K_m_ = 24 μM) and converts it efficiently. The substrate spectrum of rPsaDyP also includes the substituted phenols DMP and guaiacol. Both of these substrates show a high K_m_ (713 and 1227 µM, respectively) and a relatively low catalytic efficiency compared to the other substrates.

**Table 1:** Apparent kinetic constants of the recombinant DyP-type peroxidase from *Pleurotus sapidus*

Substrate	pH	Enzyme concentration (nM)	*K*_m_ (µM)	*k*_cat_ (s^−1^)	*k*_cat_ *K*_m_^−1^ (s^−1^ M^−1^)	*v*_max_ (µM s^−1^)
ABTS	3.8	0.27	99	375	3.8 × 10^6^	0.10
DMP	4.5	1.8	1227	60	4.9 × 10^4^	0.11
Guaiacol	4.5	4.5	713	74	1.0 × 10^5^	0.35
RBlue 5	4.0	5.4	24	18	7.5 × 10^5^	0.10

DyP is also able to catalyze the degradation of the natural pigment *β*-carotene ene (90 U L^−1^). A study of the kinetics of the conversion of this substrate was difficult, however, since only low substrate concentrations (maximal 26 µM) could be used in the assay and thus saturation of the enzyme could not be achieved. Notably, the DyP-type peroxidase can convert this substrate without the addition of H_2_O_2_ in the same manner as MsP1 and MsP2 (Scheibner et al. 2008; Zorn et al. 2003b). At the same time, enrichment of the buffer with O_2_ increased the conversion rate by a factor of 2.3 compared to standard conditions without addition of H_2_O_2_. The enzymatic conversion was virtually halted when degassed buffer was used (Table 2). This indicates that the enzyme also possesses oxidase or oxygenase activity.

**Table 2:** Degradation of *β*-carotene by the purified recombinant DyP-type peroxidase from *Pleurotus sapidus* (in the absence of H_2_O_2_) under standard assay conditions, in oxygen saturated buffer, and under anoxic conditions

Enzyme concentration (nM)	*v* (µM min^−1^)		
	Degassed buffer	Standard assay conditions	Oxygen saturated buffer
270	0.045	0.385	0.878
360	0.056	0.495	1.069
540	0.077	0.532	1.217

Furthermore, the enzyme can also convert additional natural pigments such as bixin and annatto (90 and 114 U L^−1^), as well as high redox potential dyes such as RBlack 5 (231 U L^−1^).

## Discussion

The first published sequence of a DyP-type peroxidase originated from the Basidiomycetes *B. adusta* (Kim and Shoda 1999b). In the meantime further DyP-type peroxidases from white-rot fungi have been cloned and sequenced, including *P. ostreatus*, *M. scorodonius* und *A. auricula*–*judae* (Faraco et al. 2007; Scheibner et al. 2008; Liers et al. 2010). X-ray structural analysis has only been performed on two DyP-type peroxidases from fungi. The first DyP-peroxidase structure examined was from *B. adusta* (BadDyP; PDB-Code 2D3Q). In the meantime the structure of a DyP from *A. auricula*-*judae* (AauDyP; PDB-Code 4AU9) has also been elucidated. In the present study the first DyP-type peroxidase from *P. sapidus* was cloned and sequenced. By comparing the converted amino acid sequence with other DyP-type peroxidases, and with the structural homology model of the structure of AauDyP as a basis, shared motifs and catalytic residues could be identified. The model demonstrates the characteristic *β*-barrel structure and environment of the heme pocket. This includes the characteristic GxxDG motif with the conserved aspartate that, together with the conserved arginine residue is situated on the distal side of the heme, as well as the proximal histidine. The alignment shows that the amino acids involved in the heme binding as well as those involved in catalysis are highly conserved.

DyP-type peroxidases are also capable of oxidizing large substrates that are not able to reach the immediate proximity of the heme in the active center. For this reason it is a matter of discussion as to whether these enzymes exclusively possess solvent-exposed substrate binding sites (Liers et al. 2013a). Strittmatter et al. (2013) identified potential LRET transfer pathways in the DyP-type peroxidase from *A. auricula*-*judae.* A number of exposed residues on the protein surface (Trp or Tyr) serve as oxidation site for large substrates. Linde et al. (2015a, b) recently showed that LRET from AauDyP essentially begins at W377 that is also conserved in rPsaDyP.

The histidine 164 or 166 (BadDyP) are conserved in many DyP sequences. It was therefore long a matter of discussion whether these residues function as proximal histidine or as heme ligand. Sugano et al. (2004) showed that H166 is not essential for the peroxidase activity. On the other hand, the authors showed that mutant H164A completely lost the activity. This result indicates that H164 is not directly involved in heme binding, but rather suggests only a decrease in protein stability and a loss of the heme-binding affinity (Faraco et al. 2007). H164 is not conserved in rPsaDyP or in PosDyP, but is replaced by a lysine (K167). This exchange is also found in various other representatives of the DyP-type peroxidase family, for instance in the proteins of *A. oryzae* (Q2UPE9, Q2U1I3), *Neurospora crassa* (Q7S3A4) and various other members of the DyP-family (Faraco et al. 2007). This suggests that H164 is not directly involved in heme binding and another residue coordinates the heme in DyP-type peroxidases. Nonetheless, according to Sugano (2009) H164 plays an important role in the folding of DyP-type peroxidases and binding of heme, even if it is not conserved in all members of this protein family. Here the situation is different than described by the authors. Histidine does not appear to be crucial for folding, but rather, a basic amino acid in this position. Johjima et al. (2003) identified 10 potential ligands (His, Tyr und Cys) for heme. For AauDyp Strittmatter et al. (2013) showed that on the proximal side arginine 255 and 311 form hydrogen bonds to the propionate residues of heme and are involved in the coordination of heme. The homologous residues from rPsaDyP were identified (at position 267 and 324) and in the model are at a distance of 3.2 and 3.5 Å, respectively, to the propionates.

### Heterologous expression

The DyP-type peroxidase from *Pleurotus sapidus* was successfully expressed heterologously in the ascomycete *T. reesei* and the active enzyme was secreted into the culture supernatant. An activity of 55,000 U L^−1^ was determined for the recombinant DyP-type peroxidase in the supernatant with the substrate ABTS. Heterologous expression of the DyP from *B. adusta* in *A. oryzae* imparted a 42 fold increase in activity with the substrate RBlue 5 (8 × 10^2^ U L^−1^) over that in the culture medium (Sugano et al. 2000). Heterologous expression of the PsaDyP from *T. reesei* led to an order of magnitude higher activity (5 × 10^3^ U L^−1^) with RBlue 5. PsaDyP could be efficiently expressed in *T. reesei* and unusually high activities could be achieved. The recombinant enzyme showed the characteristic absorption maximum at 409 nm (Soret–Band) and two further maxima (α and β) in the region of 640 nm that are attributed to the porphyrin structure of heme (Glenn and Gold 1985; Renganathan and Gold 1986).

### Native conformation

A glycosylation degree of 9% was determined for the DyP-type peroxidase heterologously expressed in *T. reesei,* a carbohydrate content that is comparable with that of the DyP-type peroxidase MsP2 (Scheibner et al. 2008). The carbohydrate content of DyP-type peroxidases lies typically between 9 and 31% (Hofrichter et al. 2010).

After deglycosylation the apparent molecular weight determined in SDS-PAGE was lower for rPsaDyP heterologously expressed in *T. reesei* than was calculated from the primary sequence. This implies that the enzyme, just as with BadDyP in *A. oryzae* (Sugano et al. 2000), is further processed in the host organism, *T. reesei*. The N-terminus of the recombinant enzyme was sequenced by Edman degradation. Processing of rPsaDyP is between amino acids 62 and 63. Sugano et al. (2000) showed that the recombinant DyP-type peroxidase from *B. adusta* produced in *A. oryzae* has the same N-terminus as the wild-type enzyme. This suggests that the recombinant and native PsaDyP have the same N-terminus. Studies of other DyP-type peroxidases show that processing occurs between position 56/57 (BadDyP), 55/56 (MsP1), 57/58 (MsP2) and 61/62 (AauDyP) (Liers et al. 2010; Scheibner et al. 2008; Sugano et al. 2000). In rPsaDyP a cluster of conserved amino acids is present in the region of the N-terminus, however a typical cleavage site was not found (Liers et al. 2010).

In contrast to the classical DyP rPsaDyP occurs as a dimer, whereby it must be noted that various other DyP-type peroxidases, especially from prokaryotes, form numerous higher quaternary structures ranging from monomers to hexamers. The reason for this oligomerization has been subject of discussion, but remains unknown. Sugano (2009) showed that the classical DyP, compared to DyP-type peroxidases that form oligomers, exhibit insertions in the primary sequence that are missing in the former. Nonetheless, the primary sequences of MsP1 and MsP2 exhibit a high degree of homology to BadDyP and like rPsaDyP and have these insertions, and yet they occur natively as dimers (Scheibner et al. 2008).

### Biochemical characterization

DyP-type peroxidases are typically secreted glycoproteins with molecular weights of 40–67 kDa (in monomeric form) and isoelectric points in the acid range (3.5–4.3, Hofrichter et al. 2010). A monomer of rPsaDyP has a molecular weight of 57.4 kDa and therefore is of average size for an enzyme of the family of DyP-type peroxidases. In contrast to other enzymes of this family rPsaDyP has an apparent Pl within the neutral range. Lignolytic peroxidases from fungi (LiP, VP, DyP), like plant peroxidases demonstrate maximum activity in an acidic milieu (pH 1.5–5.0, Camarero et al. 1999; Gazarian et al. 1996; Liers et al. 2010; McEldoon et al. 1995). Depending upon the substrate, the pH profile of rPsaDyP shows an activity maximum between pH 3.5 and 4. 5 (with the exception of Annatto at pH 6.0). Thus, the profile is shifted to somewhat higher pH values compared to other DyP-type peroxidases. Maximum activity of rPsaDyP was shown between 15 and 30 °C. This is comparable with that of BadDyP (Kim and Shoda 1999a). Above 35 °C activity of rPsaDyP decreased continuously and ~65% remained at 50 °C. rPsaDyP is active in a wide range of temperatures and pH values. It remains active for a number of hours in sodium acetate buffer between pH 3 and pH 6 and maintains up to 60% of its initial activity after 24 h (data not shown). There is a tendency for the activity of the enzyme to remain stable at higher pH values. After 24 h under reaction conditions it retains more than 40% of its initial activity (data not shown).

### Influence of hydrogen peroxide on enzyme activity

With ABTS as substrate the peroxidase activity dropped significantly if the H_2_O_2_ concentration rose above 0.125 mM. Inhibition of peroxidase activity by an excess of hydrogen peroxide via suicide inactivation has long been known (Arnao et al. 1990), but has not been fully resolved for DyP-type peroxidases. Maximal activity was achieved at an H_2_O_2_ concentration of 0.125 mM. For the substrates Rblue 5, DMP, guaiacol and RBlack 5 activity was inhibited at lower H_2_O_2_ concentrations. The inhibition of the enzyme activity strongly depends upon enzyme and substrate concentration. Kim and Shoda (1999a) demonstrated that the degree of inhibition varied greatly depending on the substrate used. In previously described inhibition pathways Compound II plays an important role, however, the existence of Compound II has not been confirmed in DyP-type peroxidases (Hofrichter et al. 2010; Sugano et al. 2007). The catalytic cycle described for classical peroxidases in the presence of reducing substrates begins with the oxidation to Compound I by the transfer of two electrons to H_2_O_2_. By the transfer of a single electron from the reduced substrate Compound II is formed, which in turn is reduced to the native enzyme through reaction with an additional substrate molecule. In the presence of excess H_2_O_2_ Compound II reacts with H_2_O_2_ to form Compound III (inactive form). This does not necessarily imply a final exclusion of the enzyme from the catalytic cycle. In the case of horseradish peroxidase there are indications that the enzyme slowly returns to the initial state through spontaneous decay of Compound III, giving rise to a superoxide. Furthermore, Compound III can be reduced to Compound I by various electron donors, allowing it to re-enter the catalytic cycle (Dequaire et al. 2002). Koduri and Tien (1995) showed that the substrate guaiacol or the phenoxyl radical were only partially able to transform Compound III to the initial state and are considerably less efficient at this process than veratryl alcohol. Dequaire et al. (2002) presented similar results for horseradish peroxidase. Compound III was reduced to Compound I by various electron donors. A similar substrate dependent mechanism may explain the divergent inhibition of the enzyme by hydrogen peroxide in rPsaDyP.

### Catalytic properties

From the functional standpoint the heterologously expressed peroxidase of *P. sapidus* can be assigned to the class of DyP-type peroxidases as based on its substrate spectrum and the efficient oxidation of RBlue 5. The enzyme binds RBlue 5 with the highest affinity (*K*m = 24 μM) of all the substrates studied. The affinity to this substrate is comparable with the affinity of DyP-type peroxidase from *B. adusta* (54 μM) and *A. auricula*-*judae* (23 μM) (Kim and Shoda 1999a; Liers et al. 2010). Although the binding affinity to the dye RBlue 5 by rPsaDyP is comparable, the decolorization rate of 7.5 × 10^5^ s^−1^ M^−1^ indicates a sixfold lower catalytic efficiency than for BadDyP and AauDyp (4.8 × 10^6^ s^−1^ M^−1^ and 5 × 10^6^ s^−1^ M^−1^, respectively).

The affinities of rPsaDyP for ABTS and unsubstituted phenols are lower. Nonetheless, the highest activity and catalytic efficiency was found for ABTS. The catalytic efficiency of rPsaDyP is 3.8 × 10^6^ s^−1^ M^−1^ and is therefore comparable to the catalytic efficiency of the DyP-type peroxidase from *Irpex lacteus* (IlaDyP) (8.0 × 10^6^ s^−1^ M^−1^; Salvachúa et al. 2013) and lower than that of AauDyP (1.8 × 10^7^ s^−1^ M^−1^; Liers et al. 2010) and MscDyP (6.3 × 10^7^ s^−1^ M^−1^; Szweda et al. 2013). The affinity of rPsaDyP is, however, also somewhat lower than the affinities of AauDyP and MscDyP to ABTS.

The substituted phenols DMP and guaiacol, which serve as classical substrates for MnP, are oxidized by rPsaDyP. The turnover number *k*_cat_ for the oxidation of DMP (*k*_cat_ = 60 s^−1^) or guaiacol (*k*_cat_ = 74 s^−1^) are comparable with those of other DyP-type peroxidases (AauDyP: *k*_cat_ = 90 s^−1^ and IlaDyP: *k*_cat_ = 70 s^−1^; Liers et al. 2010; Salvachúa et al. 2013) and manganese peroxidase (Bad MnP for DMP: *k*_cat_ = 70 s^−1^; Wang et al. 2002) and higher than those for lignin peroxidases (DMP: *k*_cat_ = 27 s^−1^, guaiacol: *k*_cat_ = 38 s^−1^; Ward et al. 2003) and the versatile peroxidases (for the oxidation of DMP without Mn(II): VP from *Pleurotus eryngii*: *k*_cat_ = 3 s^−1^ or BadVP: *k*_cat_ = 2.3 s^−1^; Camarero et al. 1999; Mester and Field 1998). The *K*_*m*_-values for rPsaDyP are relatively high compared to DyP–type peroxidases so that catalytic efficiency is about a fold lower.

Rblack 5 is a dye and a specific substrate for VP (Camarero et al. 1999; Heinfling et al. 1998). Liers et al. (2013b) showed that a number of DyP-type peroxidases oxidize Rblack 5. rPsaDyP oxidizes RBlack 5 at low efficiency (0.1 U mg^−1^).

Degradation of *β*-carotene and annatto (a dye mixture of the xanthophylls bixin und norbixin) was demonstrated using the purified enzyme. An aqueous-alkaline extract, the principle component of which was the sodium salt of norbixin, was used for the oxidation of annatto (Scotter et al. 1998). The degradation of bixin was also used to determine whether rPsaDyP can oxidize both xanthophylls. The enzyme also oxidizes bixin. It should be noted that higher activities were determined for norbixin (as an aqueous-alkaline annatto extract) at lower substrate concentrations (15–19 μM). A comparable activity was measured for the oxidation of the hydrophobic substrates bixin and and *β*-carotene at the same substrate concentrations (19 μM).

rPsaDyP can oxidize *β*-carotene without addition of H_2_O_2_, however enzyme activity was enhanced by adding H_2_O_2_. Enrichment of the reaction buffer with O_2_ also increased the transformation of *β*-carotene, and conversely, degassing the buffer slowed down the reaction. This indicates that the decomposition of *β*-carotene is directly dependent upon the concentration of molecular oxygen in the buffer. In addition, this implies that the in addition to the peroxidase function there is an oxidase- or oxygenase function (Sugano 2009). Zorn et al. (2003a) also showed that *β*-carotene is degraded in an oxygen-dependent reaction by a cell-free supernatant of a *Mycetinis scorodonius* culture. In other studies Scheibner (2006) and Hülsdau (2007) showed that the oxidation of *β*-carotene by the purified DyP-type peroxidase MsP1 from culture supernatant can take place without addition of H_2_O_2_, whereby the enzyme activity was increased by addition of H_2_O_2_. An H_2_O_2_ independent reaction was described for the oxidation of epinephrine by horseradish peroxidase or for the oxidation of Indol-3-acetic acid by plant peroxidases (Adak et al. 1998; Gazarian et al. 1998). Here, an autocatalytic process was suggested in which superoxide radicals are formed in the presence of molecular oxygen. These can in turn form peroxides that can be utilized by the peroxidases (Adak et al. 1998; Gazarian et al. 1998).

Due to their versatility DyP-type peroxidases may, in addition to the degradation of lignocelluloses and other polymers, take part in an unspecific pathogen defense and in detoxification processes (Liers et al. 2013b; Zámocký et al. 2009).

## Abbreviations

AA: amino acid
ABTS: 2,2′-azino-bis(3-ethylthiazoline-6-sulfonate)
DMP: dimethoxyphenol
DyP-tyxpe peroxidase: dye decolorizing peroxidase
FPLC: fast protein liquid chromatography
GFC: gel filtration chromatography
IEF: isoelectric focusing
LiP: lignin peroxidases
LRET: long range electron transfer
MnP: manganese peroxidases
MscDyP: recombinant dye decolorizing peroxidase cloned from *Mycetinis scorodonius*
PAGE: polyacrylamide gel electrophoresis
pl: isoelectric point
Rblack 5: Reactive Black 5 dye
Rblue 5: Reactive Blue 5 dye
rPsaDyP: recombinant dye decolorizing peroxidase cloned from *Pleurotus sapidus*
TMB: 3,3′,5,5′-tetramethylbenzidine
VP: versatile peroxidases
